# A Popular Treatise on the Teeth

**Published:** 1845-12

**Authors:** 


*A Popular Treatise on the Teeth;* embracing a description of their structure, the diseases to which they are subject, and their treatment, both for the prevention and cure of those diseases; together with an account of the useful methods of inserting Artificial Teeth. By ROBERT ARTHUR, Doctor of Dental Surgery, and Fellow of the American Society of Dental Surgeons. NEW YORK: E. FERRETT & Co. pp. 187, 1845.

We have always been an advocate for the diffusion of correct information on the means for the prevention and cure of the diseases of the dental apparatus. With this knowledge, every individual should be familiar, not that every one would thereby be enabled to apply the remedies for the cure of these diseases, but that they might often prevent their occurrence, and when they do occur, that in seeking professional aid, they might be enabled to discriminnte between the skilful practitioner and the empiric. Entertaining these views, it gives us pleasure to announce the publication of the above named work, which we regard, so far as we have had an opportunity of examining it, as one of the best popular treatises, upon the subject extant.

It is evident from the manner in which Dr. Arthur treats the subjects embraced in this little volume, that he is familiar with them. His views also appear to be correct, which in a popular treatise, is of vast importance, for the reason, that the general reader would not be so likely to detect false doctrines, as the professional dentist. It contains much excellent advise, and many valuable directions with regard to the means for the preservation of the teeth, and the health of the mouth, while it at the same time enters sufficiently into scientific and practical detail for all the purposes for which it is designed. The work is neatly gotten up, and as a literary production, is highly creditable to the author.

